# Intercropping Alters Phytochemical Defenses Against Insect Herbivory

**DOI:** 10.21203/rs.3.rs-4920649/v1

**Published:** 2024-09-09

**Authors:** JARROD Q. FYIE, CHASE A. STRATTON, WILLIAM R. MORRISON, EBONY G. MURRELL

**Affiliations:** The Land Institute; The Land Institute; Agricultural Research Service - Plains Area; The Land Institute

**Keywords:** Agroecology, Phytochemistry, VOCs, GC-MS, Intercropping, Herbivory

## Abstract

Given the multiple possible mechanisms for interspecific chemical interaction between adjacent heterospecific plants, phytochemical defenses of pest-susceptible crop species could potentially be enhanced or altered by intercropping with phytochemically diverse neighbors. We assessed the influence of intercropping between phytochemically diverse plants on aerial volatile organic compound (VOC) emission profiles by intercropping *Melilotus alba* and Triticum *aestivum* with *Silphium integrifolium* in AMF-inoculated soil. We also assessed the impact of intercropping on induced plant defenses by conducting an in-situ, no-choice bioassay with *Spodoptera frugiperda*. Of eight compound classes we identified across the three plant species, prenol lipids (terpenoids) were upregulated in silflower plants when monocropped with wheat and when herbivory was induced. Carboxylic acids and organooxygen compounds were reduced in sweetclover when intercropped with silflower, but increased under herbivory. Uninfested wheat plants emitted more organooxygen compounds and fatty acyls than infested plants when intercropped with silflower, but not when monocropped. This study showed that VOC emissions of plants from three diverse taxa are altered by both intercropping and herbivory in ways that may impact their resistance to insect herbivory. Further research into the role of intercropping on pest resistance in agroecological systems could help farmers to design intercropping systems that optimize natural plant herbivory defenses, thus improving agricultural sustainability.

## Introduction

Plants produce an incredibly diverse array of secondary metabolites (phytochemicals) that influence insect communities (repelling herbivores, alerting predators, attracting pollinators, and other roles) (Salazar et al. 2018; Kessler and Kalske 2018; Wetzel and Whitehead 2020; Paul et al. 2021). Since insect herbivore success is affected by both alpha (within plant) and beta (between plant) diversity, the chemical composition within individual plants and between neighbors should impact pest dynamics in the field (Wetzel and Whitehead 2020; Glassmire et al. 2020). Plant-plant communication can happen above- and belowground but chemical diversity and plant-plant interactions are complex and difficult to predict (Glinwood et al. 2011; Babikova et al. 2013; Ninkovic et al. 2013; Jing et al. 2021). Deciphering the chemical mechanisms behind plant-plant and plant-insect interactions can help develop intercropping arrangements that maintain crop productivity while minimizing inputs (e.g., pesticides) in modern agroecological systems (Silva et al. 2018).

The complexity of phytochemical expression in diverse agricultural landscapes is likely a key mechanism driving reduced pest pressure, and thus less insecticide application, compared to simplified monoculture landscapes (Bianchi et al. 2006; Haddad et al. 2009; Meehan et al. 2011; Silva et al. 2018; He et al. 2019; Paredes et al. 2021). Within-species communication is well-described in multiple contexts, with direct routes of access to physiological effects on neighbors (Kost and Heil 2008, Babikova et al. 2013, Moreira and Abdala-Roberts 2019). For example, when exposed to the odors released by beetle-damaged lima bean shoots, undamaged conspecifics expressed more defensive molecules to reduce herbivory, while also increasing extrafloral nectar production to attract natural enemies (Heil 2004; Kost and Heil 2008). While much is known about defensive priming by conspecifics, less is understood about heterospecific communication and consequent impacts on herbivore defense (but see Karban et al. 2000; Himanen et al. 2010). This is likely due to the complex and context-specific nature of these interactions.

Neighborhood dynamics are important to consider when developing agroecological systems that are optimized for defensive phytochemical production (Jing et al. 2021). Focusing on the commonalities and/or distinctions between diverse plant chemistries could provide new directions for assessing functional potential of different species, facilitating the selection of beneficial plant pairings. While every plant species produces a unique phytochemical array, the molecules are composed of common subunits that emerge from related biosynthetic pathways (Dudareva et al. 2004; Pichersky et al. 2006; Noushahi et al. 2022). The molecules that volatilize from plant tissue link them to external elements of their ecology and can be potent in their effect (Dicke 2000; Meiners 2015; Dyer et al. 2018; Hartmann 2004). For instance, push-pull systems use a chemically repellant intercrop (such as *Desmodium spp*.) to “push” pest insects away from the cash crop, while a more chemically attractive host species is planted nearby to “pull” in the pests for mechanical elimination (Cook et al. 2007; Pickett et al. 2014; Ben-Issa et al. 2017). Another tactic is intercropping cash crops with plants that chemically attract natural enemies (Ben-Issa et al. 2017).

Although phytochemically-mediated interactions tend to be species-specific and vary according to many diverse conditions, different functional categories may have predictive value for the quality of interaction (DeHaan et al. 2010; Stratton et al. 2019; Whitehead et al. 2021; Zhang et al. 2021). As these shared chemistries are understood, they can be used to regulate external factors that may influence plant success (e.g. increasing the concentration/diversity of semiochemicals to modify pest insect behavior). Adding layers of biological and chemical information to polyculture compatibility trials paves a new direction for a generalizable understanding of functions that plants may contribute in an agroecological context. Furthermore, this type of comparative framing provides a broader synthesis of the basic ecological relationships that emerged across the evolutionary history of land plants.

In this study, we tested phytochemical responses to intercropping and herbivory of three major herbaceous crop families: asters (Asteraceae), legumes (Fabaceae), and grasses (Poaceae). *Silphium integrifolium* (silflower; Asteraceae) is a North American grassland plant that is being bred as a novel perennial oilseed, cattle forage, and honey crop (Vilela et al. 2020; Schiffner et al. 2021). Compared to annual sunflower (*Helianthus annuus*), silflower has high resistance to generalist insect herbivores, likely due to its complex suite of defensive phytochemical compounds (Fiedler and Landis 2007; Kowalska et al. 2020). As a perennial prairie plant (Cassetta et al. 2023), it is also adapted to growing alongside multiple grass and legume species. White sweetclover (*Melilotus albus*, Fabaceae) and wheat (*Triticum aestivum*, Poeaceae), are forage and grain crops, respectively, that commonly grow in the same regions (Tilley et al. 2008, Zabala et al. 2018, Singh et al. 2023). We predicted that the volatile profiles of individual species would differ based on the species of their companion plant, and these differences would be more pronounced when each species was challenged by an herbivore. Specifically, we expected qualitative and quantitative increases in defense compounds with diverse plantings experiencing herbivory.

## Methods And Materials

### Experimental design

A greenhouse biculture experiment was designed to test the effect of intercropping on phytochemistries of the three species. In preliminary studies (unpublished data), neither wheat nor sweetclover demonstrated competitive growth interactions when planted in biculture with silflower. The inclusion of an aster (silflower), a legume (sweetclover), and a grain-producing grass (wheat) covers key agriculturally relevant taxa.

Silflower seeds were selected from an open-pollinated breeding population at The Land Institute, Salina, KS. White sweetclover seed was purchased from Outsidepride Seed Source, LLC (Independence, OR). Wheat seed (spring hard red variety) was purchased from True Leaf Market Seed Company (Salt Lake City, Utah). Each plant was grown from seed in individual peat moss pots. On 7 April 2021, silflower was treated with 10% bleach and 2-week cold (5°C) stratification prior to planting (Reinert et al. 2018). On 4 May 2021, white sweetclover was treated with 5-days in a dry oven at 60°C prior to planting (Gucker 2009). On 12 May 2021, spring wheat was directly seeded without additional treatment.

On 24 May 2021, seedlings with 1–2 true leaves were transplanted into oval polypropylene plastic pots (20 cm × 40 cm × 15 cm) (HC Wholesale Plant Containers, Twinsburg, OH). The pots were filled with Pro-Mix^®^ BX Biofungicide + Mycorrhizae^™^ potting soil mix (Premier Tech Horticulture, Quakertown, PA). Each plant was placed approximately 20 cm apart within the pot. 15 mL of MycoBloom^®^ mycorrhizae inoculum was added to each transplanting hole to ensure AMF establishment (Mycobloom, Lawrence, KS). Two Osmocote Plus^®^ fertilizer pellets (11-8-15 NPK) were added to the pots—one for each plant—on the side farthest from the companion plant. Plants were then grown in greenhouse conditions (23.8°C, 16:8 light:dark cycle) and watered daily for seven weeks. In total, there were 25 pots of each biculture treatment (silflower-wheat, and silflower-sweetclover) and 15 pots of each of the three monoculture treatments (silflower-silflower, wheat-wheat, and sweetclover-sweetclover). Pots from each treatment were equally divided and arranged in a randomized block design with 5 blocks. Plants were treated following basic greenhouse pest control procedures using soil nematodes for fungus gnats and Aria^®^ (flonicamid) for thrips (FMC Corporation, Philadelphia, PA), but otherwise were not exposed to chemical treatments.

### Data collection

After 6 weeks, volatile chemical emissions (VOCs) were collected from two randomly selected plants of each species by treatment combination. One week later, an in-situ bioassay was performed with *Spodoptera frugiperda* larvae (fall armyworm) to test constitutive defense of individual plants by cropping treatment. At the time of the bioassay, all sweetclover plants were flowering, but no wheat or silflower plants were.

Fall armyworm was chosen for the herbivory treatment because it is a major generalist pest of multiple field crops, including both grasses and legumes (Overton et al. 2021), and it will also feed on *Silphium integrifolium* (Peterson et al. 2022). Second instar *S. frugiperda* were purchased from Frontier Scientific Service Agriculture (Newark, DE). Larvae of approximately the same size were paired and weighed prior to placement on randomly selected plants.

For the bioassay, pots were randomly selected (15 of each biculture treatment and 7 of each monoculture treatment) and both plants in each selected pot were infested. On 13 July 2021, two *S. frugiperda* larvae were placed on 15 plants of each species in biculture and 14 plants of each species in monoculture. Larvae were contained using plastic mesh bags (37.5 cm × 23 cm) tied at the base of each plant. Mesh bags were also placed on the remaining uninfested plants as a control. After four days, fall armyworm larvae were removed. On 18 July 2021, one day after the larvae were removed, VOCs were collected from two infested and two uninfested plants of each species and treatment combination.

For each plant, VOCs were collected from the headspace using custom field aeration kits for 2-hour intervals. Plants were sealed in non-reactive polyethylene terephthalate (PET) bags (24 cm × 33 cm) attached to glass-plastic tubes filled with polydivinylbenzene adsorbent. Tubes were stored in aluminum foil at −80°C until they were eluted with 500 μL of dichloromethane and purged with inert helium gas. Eluted samples were stored at −80°C in 2 mL glass vials with 250-μL glass inserts with polymer feet. A total of 2 μL (381 ng) of tetradecane internal standard was added to 200 μL subsamples of each sample prior to gas chromatography coupled with mass spectrometry (GC-MS) analysis.

To test for the presence of any growth-defense tradeoffs (Monson et al. 2022) due to interspecific competition, all plants were destructively harvested on 19 July 2021 for aboveground biomass. Biomass was dried at 6.7°C and 9% relative humidity for 2 weeks and then weighed.

### Chemical identification and classification

GC-MS analysis was performed on an Agilent 7890B gas chromatograph (GC) (Agilent Technologies, Inc., Santa Clara, CA, USA) equipped with an Agilent Durabond HP-5 column (30 m length, 0.250 mm diameter and 0.25 μm film thickness) with helium as carrier gas at a constant 1.2 mL/min flow and 40 cm/s velocity, which was coupled with a single-quadrupole Agilent 5977B mass spectrometer (MS) (after Van Winkle et al. 2022). Samples were injected with an autosampler in splitless mode. The compounds were separated by auto-injecting 1 μl of each sample in the inlet at 250°C, with a clean cycle of 1 μL dichloromethane, methanol, and sample in between. The oven temperature was programmed at 40°C for 1 min followed by 10°C/min increase to 300°C and then held for 26.5 min. After a solvent delay of 4 min, mass ranges between 50 and 550 atomic mass units were scanned. Top hits for all peaks were identified using Agilent’s Unknowns Analysis software matching with the NIST2020 mass spec library through deconvolution.

Top hits from NIST library matches (at 70% match factor or higher) were extracted from samples using the R package “uafR” (Stratton et al. 2024). Relevant compounds were selected by running all tentatively identified molecules through *categorate()* with a custom library that structurally matched for common plant chemistries (e.g. terpenes, phenylpropanoids, benzenoids, flavonoids, and green leafy volatiles, Supplemental Table 1). Known contaminants were also removed using output from this function. Chemical component areas for optimal compound identifications were then extracted from the samples using *exactoThese()* and *mzExacto()* then standardized relative to the internal standard (tetradecane) using *standardifyIt()* with default settings.

From the raw GC-MS data, we removed any chemicals that were found in fewer than 20% of all samples, or fewer than 30% of samples from at least one plant species. Since the number of chemicals after this reduction – 50 compounds – exceeded the number of VOC samples collected (n = 43), The abundances of the remaining 50 chemicals were then summed by structural chemical class using the web-based application ClassyFire (http://classyfire.wishartlab.com/). Forty-six of the compounds were assigned to seven chemical classes, while the remaining four compounds were combined into an eighth group (“Other”).

### Statistical analyses

All statistical analyses were done using SAS Statistical Software. The abundances of each of eight chemical classes were ln + 1 transformed to meet the assumption of normality, then analyzed using Principal Component Analysis (PROC FACTOR). The first two chemical PCs were then analyzed for each plant species using MANOVAs (PROC GLM), with the two chemical PCs as the dependent factors, and treatment, infestation, and the treatment × infestation interaction as fixed, independent factors. We also conducted ANOVAs on every chemical class for each plant species (PROC GLM), testing the fixed effects of treatment, infestation, and the treatment × infestation interaction on ln + 1 compound class abundance. Significant main effects or interactions were then further tested by calculating pairwise comparisons of least square means with a Tukey-Kramer adjustment (LSMEANS statement).

Aboveground dry biomass was analyzed for each species using two-way ANOVAs (PROC GLM), with plant species, herbivory treatment (presence vs. absence) and cropping treatment (monocrop vs. intercrop(s)) as fixed, categorical independent variables. Assumptions were tested using Shapiro-Wilks test for normality and visual plots for homoscedasticity of residuals. Post-hoc pairwise comparisons with a Tukey adjustment were conducted on significant effects/interactions.

## Results

**Figure 1:**
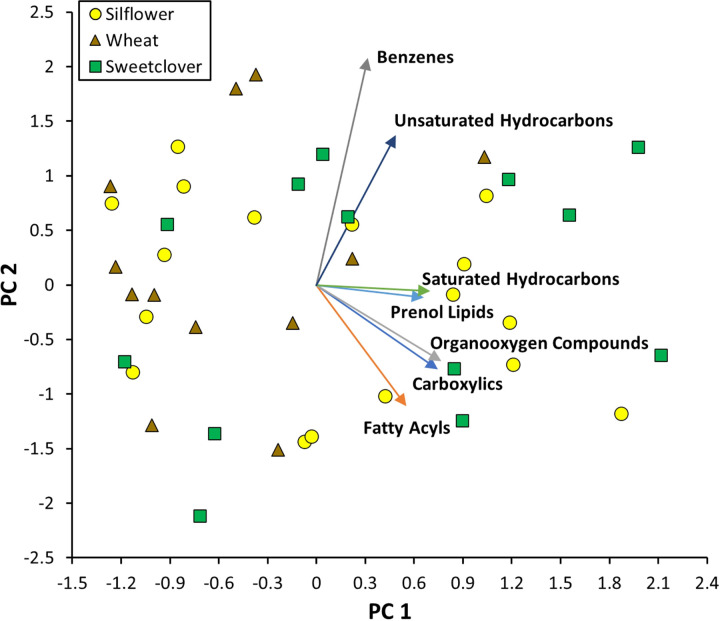
PCA analysis of compound classes (“Other” not shown) and the location of the 3 plant species in VOC ordinal trait-space. Points represent individual VOC samples, while lines show relative strength and orientation of the compound classes along both PC axes.

The uafR workflow identified 50 unique compounds from the VOC samples of each species (**Table 1**). The first two principal components (PCs) of the PCA of these 50 compounds, summed by chemical class, captured a cumulative 49.12% of the variance in the data (PC1 = 27.55%, PC2 = 21.57%). All compound classes except benzenes loaded significantly and positively on PC1. On PC2, benzenes and unsaturated hydrocarbons were positively loaded while fatty acyls were negatively loaded (Figure 1).

The MANOVA on PCs 1 and 2 found no significant main effects or interaction effect on silflower (**Table 2, Supplementary Table 1A**). Sweetclover showed significant main effects of cropping and herbivory treatments, with greater PC1 values (but not PC2) in plants that were monocropped vs. intercropped, and plants exposed to herbivory vs. pre-herbivory or control plants (**Table 2, Supplementary Table 1B**). Wheat had a significant cropping by herbivory interaction, in that intercropped control plants had higher PC1 (but not PC2) values than monocropped control plants or intercropped plants prior to herbivory (**Table 2, Supplementary Table 1B**).

**Figure 2:**
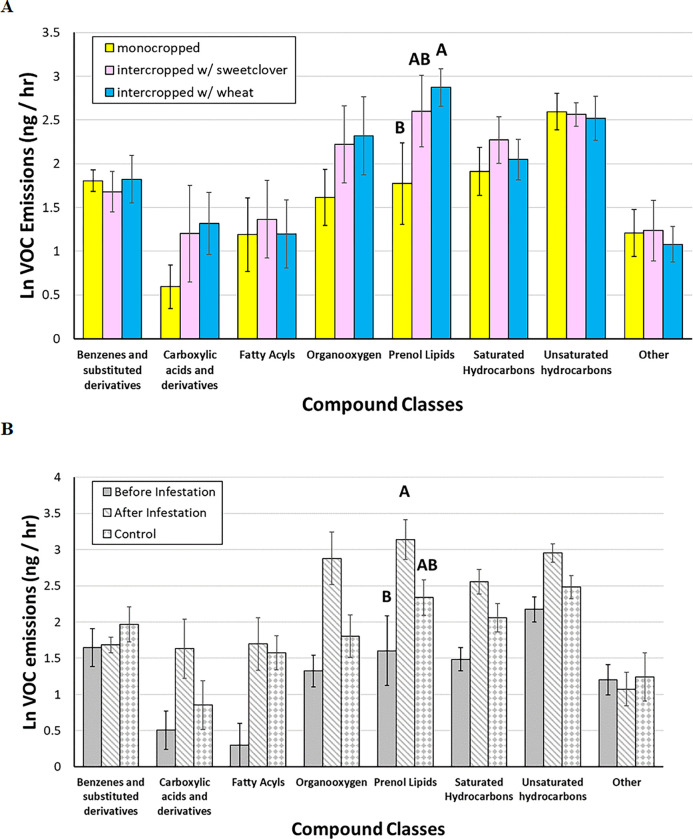
Mean ± SE of ln-transformed VOC emissions (ng/hr) of compound classes in silflower, by (a) cropping treatments, (b) herbivory treatments. Bars with letters indicate compound classes that had a significant treatment effect, and bars within those classes that share letters do not significantly differ.

**Figure 3:**
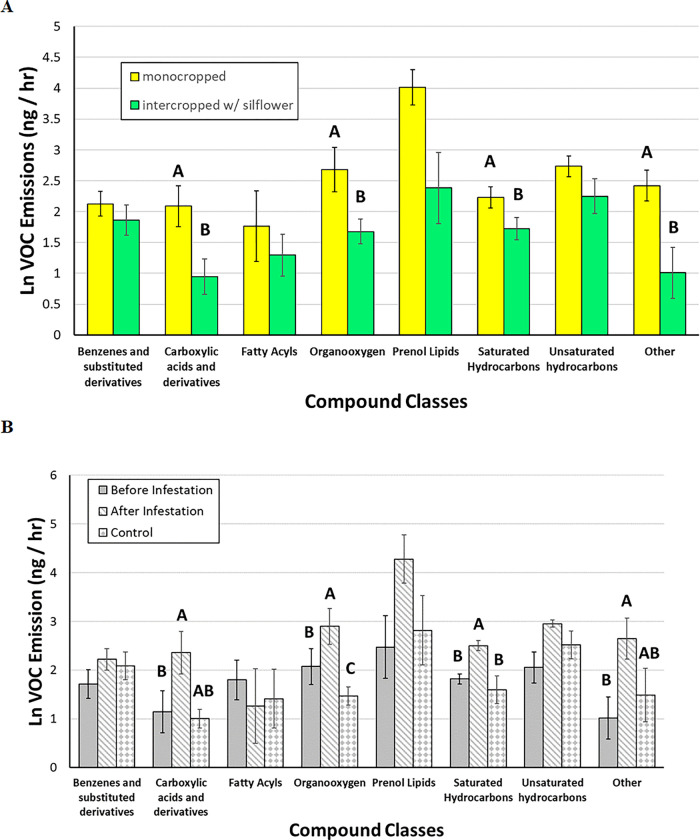
Mean ± SE of ln-transformed VOC emissions (ng/hr) of compound classes in sweetclover, by (a) cropping treatments, (b) herbivory treatments. Bars with letters indicate compound classes that had a significant treatment effect, and bars within those classes that share letters do not significantly differ.

**Figure 4:**
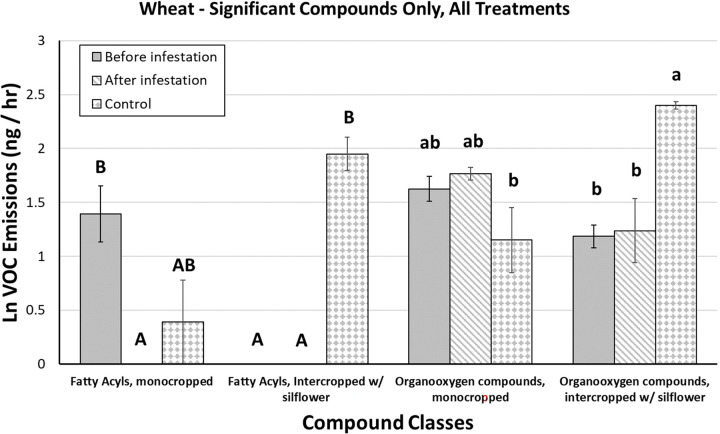
Mean ± SE of ln-transformed VOC emissions (ng/hr) in wheat, significant compounds only, across all intercropping and herbivory treatments. Bars with letters indicate compound classes that had a significant treatment effect, and bars within those classes that share letters do not significantly differ.

These ANOVAs of individual compound classes in silflower showed an increase in only the prenol lipids (terpenoids) in response to intercropping with wheat (Figure 2A) and herbivory (Figure 2B), but no interaction between these main effects (**Table 3E**). In sweetclover, multiple chemical classes shifted in response to both cropping and collection time. These include carboxylics, organooxygens, saturated hydrocarbons, and the 4 compounds grouped into “other” (**Table 3B, D, F, H**). When intercropped with silflower, sweetclover experienced a dampening in these compound classes (Figure 3A), but the same compound classes increased when challenged by fall armyworm (Figure 3B). Wheat experienced a more complicated alteration in chemical profiles with significant interaction effects in fatty acyls and organooxygens (**Table 3 C, D**). Fatty acyls were significantly higher in monocropped wheat plants before compared with after herbivory, but in intercropped plants fatty acyl levels were low both prior to and after herbivory (Figure 4). Organooxygens did not differ among monocropped treatments, but in intercropped plants they were more abundant in the control plants than in the plants exposed to herbivory, both before and after those plants were exposed (Figure 4).

**Figure 5:**
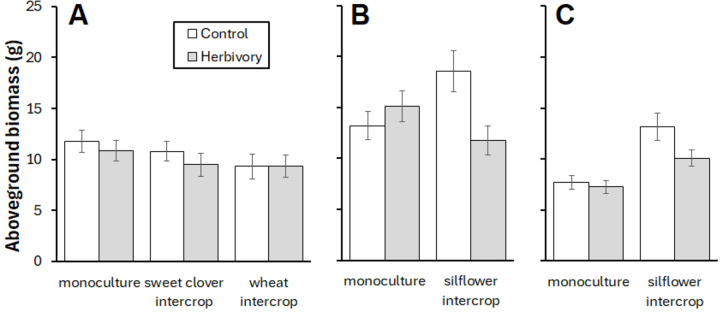
Mean ± SE aboveground biomass by species in response to intercropping and herbivory. (A) silflower, (B) sweet clover, (C) wheat.

Silflower biomass was not affected by any cropping or herbivory treatment, nor the interaction thereof (**Table 4**, Figure 5A). In sweetclover, herbivory only reduced biomass when it was intercropped with silflower (**Table 4**, Figure 5B). Wheat had increased biomass when intercropped with silflower and decreased biomass when exposed to herbivory, but there was no interaction between these two factors (**Table 4**, Figure 5C).

## Discussion

Phytochemistry is a complex and diverse trait, with unique quantitative and/or qualitative attributes for every plant species. Though the total profile is unique for each plant, similar classes/functional groupings have emerged that generalize phytochemical constituents. Using these functional groups allows comparisons of high-dimensional chemical data to occur in an ecologically relevant context. Our chemical data were described by 7 functional groups: benzene (and derivatives), carboxylic acids (and derivatives), fatty acyls, organooxygen compounds, prenol lipids (terpenoids), saturated hydrocarbons, unsaturated hydrocarbons, and “other” for 4 unclassified compounds. These groups broadly describe common patterns in phytochemical expression, allowing their shifts in different species to be compared. Our results showed that different chemical classes (and hence different biosynthetic pathways) can be altered by neighboring heterospecifics, but which compound classes are altered is species-dependent.

All the identified compound classes assessed in this study are both common in plants and relevant to plant defense. Benzene derivatives are often upregulated when plants are under stress (Misztal et al. 2015). Carboxylic acid derivatives can have major physicochemical and biological properties, acting as potent antioxidant, antimicrobial, and/or cytotoxic compounds (Godlewska-Żyłkiewicz et al. 2020). Fatty acyls are used for many cellular, physiological, and defensive roles, including membrane storage and surface lipids or the production of metabolites involved in signaling or defense (Kalinger et al. 2020). Organooxygen compounds include carbohydrates and can have antioxidant, anti-tumor, and anti-inflammatory effects (Zhou et al. 2022). Prenol lipids (terpenoids) are exceptionally diverse with varied biological activities including those for direct plant defense and attracting beneficial insects (e.g. pollinators and natural enemies) (Singh, 2015). Hydrocarbons (saturated and unsaturated) are commonly found as a protective barrier on leaves (Teoh, 2015).

Since both the biomass and phytochemical responses to intercropping and herbivory treatments were species-dependent, we will examine each species’ responses separately.

### Silphium integrifolium

In silflower, the quantity of prenol lipids (terpenoids) emitted from the leaves changed in response to both intercrop treatment and herbivore challenge. Silflower growing in biculture with wheat expressed a greater volume of terpenoids than silflower grown in monoculture and silflower grown in biculture with sweetclover expressed an intermediate amount of terpenoids. The lack of an interaction effect between the intercropping and herbivory treatments suggests that intercropping increases only silflower constitutive defenses. Notably, this increase in constitutive defenses did not appear to compromise the induced defenses of the intercropped plants.

The exact agroecological impact of these differences likely depends on the species of herbivores and natural predators in the local community because of the diverse interactions terpenes mediate. Other studies show some of the terpenes identified in this analysis have biological effects on *S. frugiperda* or other *Spodoptera spp*. Eucalyptol and α-pinene are feeding deterrents for 2nd larval instar *Spodoptera littoralis* on artificial diet (Abdelgaleil et al. 2020). Abdelgaleil et al. also showed eucalyptol suppresses larval growth and increases larval mortality of *S. littoralis*. Similarly, γ-terpinene was shown to reduce the growth of 3rd instar *S. littoralis* in a study of *Origanum vulgare* essential oil terpenes (Agliassa and Maffei 2018). Because prenol lipids have defensive properties, our data suggest that silflower growing in the presence of wheat, but not sweetclover, may be better phytochemically defended from potential pests.

Aboveground biomass results did not indicate any tradeoff between growth and chemical defense in silflower. Interestingly, there was also no measurable effect of herbivory on silflower biomass. While fall armyworm can be induced to feed on silflower in laboratory studies, in the field only its close relative *Spodoptera ornithogalli* has been recorded on this species (Prasifka et al. 2017). Feeding by fall armyworm may therefore have been reduced on this plant, only sufficing to induce a phytochemical defense response. Future studies should consider using an alternative pest species or increase the number *S. frugiperda* or how long they feed, to better test whether silflower exhibits any tradeoff between growth and phytochemical defense.

### Melilotus alba

Sweetclover also expressed different volumes of VOCs in response to both intercrop treatment and herbivore challenge, but in a different and more diverse set of compounds. Compared to when monocropped, sweetclover grown in biculture with silflower expressed lower volumes of VOCs in four classes: carboxylic acids, organooxygens, saturated hydrocarbons, and our set of four “other” compounds (see Table 1 for tentative identifications). However, after the herbivore challenge, sweetclover increased emission of these same four classes of compounds, indicating that *M. alba* uses these compounds in its defense response. Additionally, sweetclover biomass diverged when it was intercropped with silflower and then challenged with an herbivore. The combined biomass and phytochemistry data suggest a tradeoff between phytochemical defense production and growth in sweetclover when paired with silflower.

While on the surface this tradeoff may appear deleterious, sweetclover alternatively could be taking advantage of the greater semiochemical diversity of the silflower-sweetclover intercropped system to reduce the threat of herbivory to itself, as increased phytochemical diversity has been shown to reduce herbivory in plant communities (Salazar et al. 2016). Under this “phytochemical masking” hypothesis, sweetclover may respond to cues from its phytochemically divergent neighbors by reducing expression of its own signaling compounds, hiding its presence from potential herbivores within the odor plume of a well-defended neighbor. If this hypothesis is supported, one would predict that sweetclover would more likely employ this method when intercropped with chemically divergent species than with phytochemically similar heterospecifics. Field studies or behavioral assays with pests would be needed to demonstrate whether this phenomenon occurs, and if it is an effective plant defense strategy.

### Triticum aestivum

Wheat phytochemical response to intercropping and herbivory was limited to organooxygen compounds and fatty acyls. While wheat had a significant intercropping*herbivory interaction effect for these two compound classes, in neither case were these compound classes upregulated in response to either treatment. Constitutive fatty acyls were suppressed in intercropped wheat prior to infestation, but intercropped control plants (which were uninfested, but whose VOCs were collected at the same time as infested plants) had elevated fatty acyls compared to infested plants. Control plants also emitted more organooxygen compounds than infested plants when intercropped, but not when monocropped.

Wheat aboveground biomass significantly increased when intercropped with silflower, and decreased when herbivores fed on the plants, but there was no interaction effect that mimicked the pattern observed in the VOCs. Collectively, these data suggest that increased VOCs in wheat plants were linked to the greater biomass of intercropped control plants. When plants were fed upon by herbivores, the VOC emission increase that should have occurred in the infested plants (had they not been fed upon) was suppressed. Why fatty acyl content was significantly higher in uninfested vs. infested monocropped plants is unknown. However, the results from this experiment support that intercropping with silflower primarily benefits wheat growth and may only indirectly benefit (through increased growth) wheat phytochemical defense.

## Conclusion

This study shows that intercropping plants across different families can change both plant growth and VOC production in ways that may impact pest defense. The data also show that the response can be species-specific, and it provides some evidence for different growth and defense strategies that plants may employ when growing in more biodiverse assemblages. Our research demonstrates the need for further research into the role of heterospecific plant-plant interactions on pest dynamics in agroecological systems. These mechanisms could be useful in developing novel intercrop combinations that can improve plant defenses against pests and help to reduce the need for commercial pesticides.

## References

[R1] Abdelgaleil SAM, Abou-Taleb HK, Al-Nagar NMA, Shawir MS (2020) Antifeedant, growth regulatory and biochemical effects of terpenes and phenylpropenes on Spodoptera littoralis Boisduval. Int J Trop Insect Sci 40:423–433. 10.1007/S42690-019-00093-8

[R2] Agliassa C, Maffei ME (2018) Origanum vulgare Terpenoids Induce Oxidative Stress and Reduce the Feeding Activity of Spodoptera littoralis. Int J Mol Sci 19:2805. 10.3390/IJMS19092805 30231481 PMC6165561

[R3] Babikova Z, Gilbert L, Bruce TJA, (2013) Underground signals carried through common mycelial networks warn neighbouring plants of aphids attack. Ecol Lett 16:835–843. 10.1111/ele.12115 23656527

[R4] Ben-Issa R, Gomez L, Gautier H (2017) Companion plants for aphid pest management. Insects 8:112. 10.3390/insects8040112 29053585 PMC5746795

[R5] Bianchi FJJA, Booij CJH, Tscharntke T (2006) Sustainable pest regulation in agricultural landscapes: A review on landscape composition, biodiversity and natural pest control. Proc R Soc B Biol Sci 273:1715–1727. 10.1098/RSPB.2006.3530 PMC1634792 16790403

[R6] Cassetta E, Peterson K, Bever JD, Brandvain Y, Van Tassel D, Lubin TK, Alexander HM, Byers DL, Schiffner S, Turner K (2023) Adaptation of pathogens to their local plant host, Silphium integrifolium, along a precipitation gradient. Ecosphere, 14:e4565. 10.1002/ecs2.4565.

[R7] Cook SM, Khan ZR, Pickett JA (2007) The use of push-pull strategies in integrated pest management. Annu Rev Entomol 52:375–400. 10.1146/annurev.ento.52.110405.091407 16968206

[R8] DeHaan LR, Weisberg S, Tilman D, Fornara D (2010) Agricultural and biofuel implications of a species diversity experiment with native perennial grassland plants. Agric Ecosyst Environ 137:33–38. 10.1016/J.AGEE.2009.10.017

[R9] Dicke Marcel. “Chemical Ecology of Host-Plant Selection by Herbivorous Arthropods: A Multitrophic Perspective.” Biochemical Systematics and Ecology 28, no. 7 (August 1, 2000): 601–17. 10.1016/S0305-1978(99)00106-4. 10854737

[R10] Dudareva Natalia, Pichersky Eran, and Gershenzon Jonathan. “Biochemistry of Plant Volatiles.” Plant Physiology 135, no. 4 (August 1, 2004): 1893–1902. 10.1104/pp.104.049981. 15326281 PMC520761

[R11] Dyer Lee A., Philbin Casey S., Ochsenrider Kaitlin M., Richards Lora A., Massad Tara J., Smilanich Angela M., Forister Matthew L., “Modern Approaches to Study Plant–Insect Interactions in Chemical Ecology.” Nature Reviews Chemistry 2, no. 6 (June 2018): 50–64. 10.1038/s41570-018-0009-7.

[R12] Fiedler AK, Landis DA (2007) Attractiveness of Michigan Native Plants to Arthropod Natural Enemies and Herbivores. Environ Entomol 36:751–765. 10.1093/EE/36.4.751 17716466

[R13] Glassmire AE, Zehr LN, Wetzel WC (2020) Disentangling dimensions of phytochemical diversity: alpha and beta have contrasting effects on an insect herbivore. Ecology 101:. 10.1002/ecy.3158 32745232

[R14] Glinwood R, Ninkovic V, Pettersson J (2011) Chemical interaction between undamaged plants – Effects on herbivores and natural enemies. Phytochemistry 72:1683–1689. 10.1016/j.phytochem.2011.02.010 21388645

[R15] Godlewska-Żyłkiewicz B, Świsłocka R, Kalinowska M, (2020) Biologically Active Compounds of Plants: Structure-Related Antioxidant, Microbiological and Cytotoxic Activity of Selected Carboxylic Acids. Materials 13:4454. 10.3390/ma13194454 33049979 PMC7579235

[R16] Gucker CL (2009) Melilotus alba, M. officinalis. In: Fire Effects Information System, [Online]. US Dep. Agric. For. Serv. Rocky Mt. Res. Stn. Fire Sci. Lab. Prod.

[R17] Haddad NM, Crutsinger GM, Gross K, (2009) Plant species loss decreases arthropod diversity and shifts trophic structure. Ecol Lett 12:1029–1039. 10.1111/J.1461-0248.2009.01356.X 19702636

[R18] Hartmann Thomas. “Plant-Derived Secondary Metabolites as Defensive Chemicals in Herbivorous Insects: A Case Study in Chemical Ecology.” Planta 219, no. 1 (May 1, 2004): 1–4. 10.1007/s00425-004-1249-y. 15042370

[R19] He H, Liu L, Munir S, (2019) Crop diversity and pest management in sustainable agriculture. J Integr Agric 18:1945–1952. 10.1016/S2095-3119(19)62689-4

[R20] Heil M (2004) Induction of two indirect defences benefits Lima bean (Phaseolus lunatus, Fabaceae) in nature. J Ecol 92:527—536. 10.1111/j.0022-0477.2004.00890.x

[R21] Himanen SJ, Blande JD, Klemola T, Pulkkinen J, Heijari J, Holopainen JK (2010) Birch (Betula spp.) leaves adsorb and rerelease volatiles specific to neighbouring plants – a mechanism for associational herbivore resistance? New Phytol 186:722–732. 10.1111/j.1469-8137.2010.03220.x 20298484

[R22] Jing T, Qian X, Du W, (2021) Herbivore-induced volatiles influence moth preference by increasing the β-Ocimene emission of neighbouring tea plants. Plant Cell Environ 44:3667–3680. 10.1111/PCE.14174 34449086

[R23] Kalinger RS, Pulsifer IP, Hepworth SR, Rowland O (2020) Fatty Acyl Synthetases and Thioesterases in Plant Lipid Metabolism: Diverse Functions and Biotechnological Applications. Lipids 55:435–455. 10.1002/lipd.12226 32074392

[R24] Karban R, Baldwin IT, Baxter KJ, Laue G, Felton GW (2000) Communication between plants: induced resistance in wild tobacco plants following clipping of neighboring sagebrush. Oecologia 125:66–71. 10.1007/PL00008892 28308223

[R25] Kessler A, Kalske A (2018) Plant Secondary Metabolite Diversity and Species Interactions. Annu Rev Ecol Evol Syst 49:115–138. 10.1146/annurev-ecolsys-110617-062406

[R26] Kost C, Heil M (2008) The defensive role of volatile emission and extrafloral nectar secretion for Lima bean in nature. J Chem Ecol 34:2—13. 10.1007/s10886-007-9404-0 PMC2758370 18071821

[R27] Kowalska G, Pankiewicz U, Kowalski R (2020) Evaluation of Chemical Composition of Some Silphium L. Species as Alternative Raw Materials. Agric 2020 Vol 10 Page 132 10:132. 10.3390/AGRICULTURE10040132

[R28] Meehan TD, Werling BP, Landis DA, Gratton C (2011) Agricultural landscape simplification and insecticide use in the Midwestern United States. Proc Natl Acad Sci 108:11500–11505. 10.1073/PNAS.1100751108 21746934 PMC3136260

[R29] Meiners Torsten. “Chemical Ecology and Evolution of Plant–Insect Interactions: A Multitrophic Perspective.” Current Opinion in Insect Science, Ecology * Parasites/Parasitoids/Biological control, 8 (April 1, 2015): 22–28. 10.1016/j.cois.2015.02.003. 32846665

[R30] Misztal PK, Hewitt CN, Wildt J, (2015) Atmospheric benzenoid emissions from plants rival those from fossil fuels. Sci Rep 5:12064. 10.1038/srep12064 26165168 PMC4499884

[R31] Monson RK, Trowbridge AM, Lindroth RL, Lerdau MT (2022) Coordinated resource allocation to plant growth–defense tradeoffs. New Phytol 233:1051–1066. 10.1111/nph.17773. 34614214

[R32] Moreira X, Abdala-Roberts L (2019) Specificity and context-dependency of plant-plant communication in response to insect herbivory. Current C 32:15—21. 10.1016/j.cois.2018.09.003. 31113626

[R33] Ninkovic V, Dahlin I, Vucetic A, (2013) Volatile Exchange between Undamaged Plants - a New Mechanism Affecting Insect Orientation in Intercropping. PLoS ONE 8:. 10.1371/journal.pone.0069431 PMC3726678 23922710

[R34] Noushahi Hamza Armghan, Aamir Hamid Khan Usama Farhan Noushahi, Hussain Mubashar, Javed Talha, Zafar Maimoona, Batool Maria, “Biosynthetic Pathways of Triterpenoids and Strategies to Improve Their Biosynthetic Efficiency.” Plant Growth Regulation 97, no. 3 (July 1, 2022): 439–54. 10.1007/s10725-022-00818-9. 35382096 PMC8969394

[R35] Overton K, Maino JL, Day R, Umina PA, Bett B, Carnovale D, Ekesi S, Meagher R, Reynolds OL (2021) Global crop impacts, yield losses and action thresholds for fall armyworm (Spodoptera frugiperda): A review. Crop Protection 145:105641. 10.1016/j.cropro.2021.105641

[R36] Paredes D, Rosenheim JA, Chaplin-Kramer R, (2021) Landscape simplification increases vineyard pest outbreaks and insecticide use. Ecol Lett 24:73–83. 10.1111/ele.13622 33051978 PMC7756857

[R37] Paul RL, Pearse IS, Ode PJ (2021) Fine-scale plant defence variability increases top-down control of an herbivore. Funct Ecol 35:1437–1447. 10.1111/1365-2435.13808

[R38] Peterson K, Cheremond E, Brandvain Y, Van Tassel D, Murrell E (2022) Weight gain of Spodoptera frugiperda larvae (Lepidoptera: Noctuidae) on leaf and floral tissues of Silphium integrifolium (Asterales: Asteraceae) differs by plant genotype. Environ Entomol 51:397–404. 10.1093/ee/nvab146 35024830

[R39] Pickett JA, Woodcock CM, Midega CAO, Khan ZR (2014) Push-pull farming systems. Current Op Insect Sci 26:125—132. 10.1016/j.copbio.2013.12.006 24445079

[R40] Pichersky Eran, Noel Joseph P., and Dudareva Natalia. “Biosynthesis of Plant Volatiles: Nature’s Diversity and Ingenuity.” Science 311, no. 5762 (February 10, 2006): 808–11. 10.1126/science.1118510. 16469917 PMC2861909

[R41] Prasifka JR, Mallinger RE, Hulke BS, Larson SR, Van Tassel D (2017) Plant-herbivore and plant-pollinator interactions of the developing perennial oilseed crop, Silphium integrifolium. Environ Entomol 46:1339–1345. 10.1093/ee/nvx134 29029088

[R42] Reinert S, Money KL, Rockstad GBG, (2018) Two contrasting laboratory methods improve Silphium integrifolium Michx. germination rate to agronomically acceptable levels. Euphytica 2018 2149 214:1–9. 10.1007/S10681-018-2236-X

[R43] Salazar D, Jaramillo A, Marquis RJ (2016) The impact of plant chemical diversity on plant–herbivore interactions at the community level. Oecologia 181:1199–1208. 10.1007/s00442-016-3629-y 27129320

[R44] Salazar D, Lokvam J, Mesones I, (2018) Origin and maintenance of chemical diversity in a species-rich tropical tree lineage. Nat Ecol Evol 2018 26 2:983–990. 10.1038/s41559-018-0552-0 29760441

[R45] Schiffner S, Jungers JM, Tassel DV, (2021) Seeding date affects seed and biomass yield of Silphium integrifolium Michx. (silflower). Native Plants J 22:30–44. 10.3368/NPJ.22.1.30

[R46] Silva RF, Rabeschini GBP, Peinado GLR, (2018) The ecology of plant chemistry and multi-species interactions in diversified agroecosystems. Front Plant Sci 871:1–7. 10.3389/fpls.2018.01713 PMC6262048 30524464

[R47] Singh J, Chhabra B, Raza A, Yang SH, Sandhu KS (2023) Important wheat diseases in the US and their management in the 21st century. Frontiers Plant Sci 13:1010191. 10.3389/fpls.2022.1010191 PMC9877539 36714765

[R48] Stratton CA, Hodgdon E, Rodriguez-Saona C, (2019) Odors from phylogenetically-distant plants to Brassicaceae repel an herbivorous Brassica specialist. Sci Rep 2019 91 9:1–11. 10.1038/s41598-019-47094-8 31337839 PMC6650400

[R49] Stratton CA, Thompson Y, Zio K, Morrison WR III, Murrell EG (2024) uafR: An R package that automates mass spectrometry data processing. PLoS One 19:e0306202. 10.1371/journal.pone.0306202 38968199 PMC11226021

[R50] Tilley D, Ogle D, and John LS (2008). Plant guide: Yellow sweetclover & white sweetclover (Melilotus officinalis (L.) Lam. & M. alba Medik). Aberdeen, ID: US Department of Agriculture, Natural Resources Conservation Service, Aberdeen Plant Materials Center. 4 pp. Downloaded July 29, 2024 from https://plants.usda.gov/home/plantProfile?symbol=MEOF

[R51] Van Winkle T, Ponce M, Quellhorst H, Bruce A, Albin CE, Kim TN, Zhu KY and Morrison WR (2022). Microbial volatile organic compounds from tempered and incubated grain mediate attraction by a primary but not secondary stored product insect pest in wheat. Journal of Chemical Ecology, 48: 27–40. 34542783 10.1007/s10886-021-01312-8 PMC8801404

[R52] Vilela AE, González-Paleo L, Ravetta DA, (2020) Balancing Forage Production, Seed Yield, and Pest Management in the Perennial Sunflower Silphium integrifolium (Asteraceae). Agron 2020 Vol 10 Page 1471 10:1471. 10.3390/AGRONOMY10101471

[R53] Wetzel WC, Whitehead SR (2020) The many dimensions of phytochemical diversity: linking theory to practice. Ecol Lett 23:16–32. 10.1111/ELE.13422 31724320

[R54] Whitehead SR, Bass E, Corrigan A, (2021) Interaction diversity explains the maintenance of phytochemical diversity. Ecol Lett 24:1205–1214. 10.1111/ele.13736 33783114

[R55] Zabala JM, Morinoni L, Giavedoni JA, Schrauf GE (2018) Breeding strategies in Melilotus albus Desr., a salt-tolerant forage legume. Euphytica 214:22. 10.1007/s10681-017-2031-0

[R56] Zhang Y, Deng T, Sun L, (2021) Phylogenetic patterns suggest frequent multiple origins of secondary metabolites across the seed-plant “tree of life.” Natl Sci Rev 8:nwaa105. 10.1093/nsr/nwaa105 34691607 PMC8288438

[R57] Zhou D, Zhao Y, Chen Z, (2022) Traditional processing increases biological activities of Dendrobium offificinale Kimura et. Migo in Southeast Yunnan, China. Sci Rep 12:14814. 10.1038/s41598-022-17628-8 36045147 PMC9433373

